# Supplementary material for: Boosting a practical Li-CO2 battery through dimerization reaction based on solid redox mediator
Source: Nat Commun. 2024 Jan 27;15:803. doi: 10.1038/s41467-024-45087-4 (PMC11258291; doi:10.1038/s41467-024-45087-4)
Supplement: Supplementary file 1 — Supplementary Information [file 41467_2024_45087_MOESM1_ESM.pdf]

Supplementary Materials for

Boosting a practical Li-CO_2 battery through dimerization reaction based on solid redox mediator

Wei Li[1], Menghang Zhang[1], Xinyi Sun[1], Chuanchao Sheng[1], Xiaowei Mu[1], Lei Wang[1], Ping He[1,*], Haoshen Zhou[1,*]

[1]Center of Energy Storage Materials & Technology, College of Engineering and Applied Sciences, Jiangsu Key Laboratory of Artificial Functional Materials, National Laboratory of Solid-State Microstructures, and Collaborative Innovation Center of Advanced Microstructures, Nanjing University, Nanjing 210023, P. R. China

* Corresponding author: pinghe@nju.edu.cn. hszhou@nju.edu.cn

Supplementary figures and tables

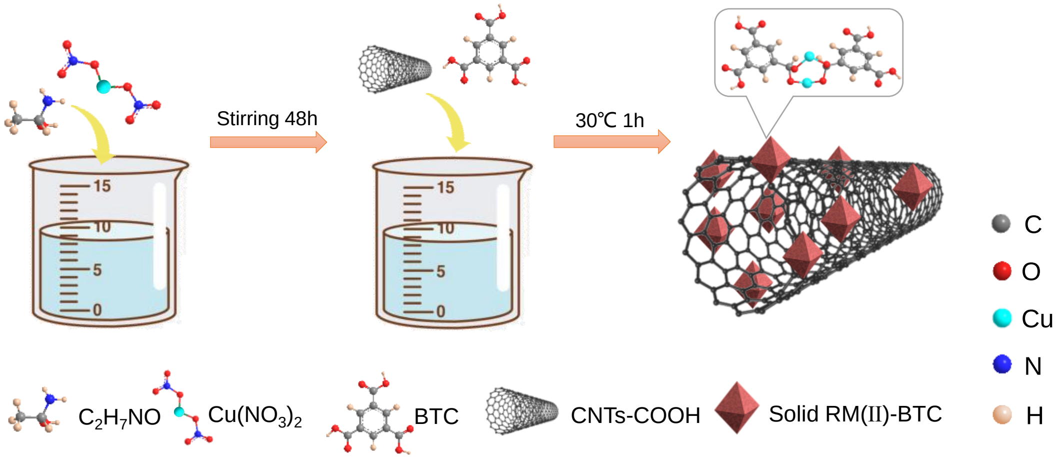

Supplementary Fig. 1. Schematics illustrating the preparation procedure of solid RM(II)-BTC cathode. The grey, red, cyan, blue, and light orange balls represent C, O, Cu, N, and H, respectively.

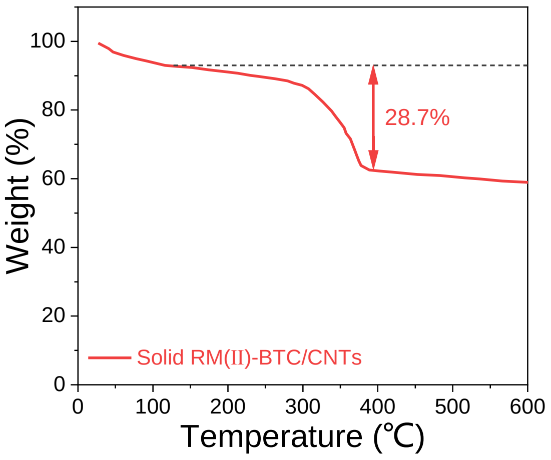

Supplementary Fig. 2. TGA curves of solid RM(II)-BTC/CNTs under N_2 atmosphere.

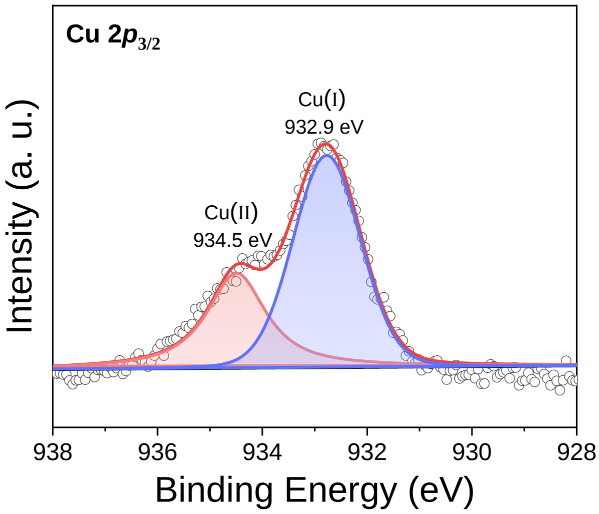

Supplementary Fig. 3. XPS characterization of Cu 2$p_{3/2}$ for deep discharged Li-CO_2 battery based on RM(II)-BTC cathode.

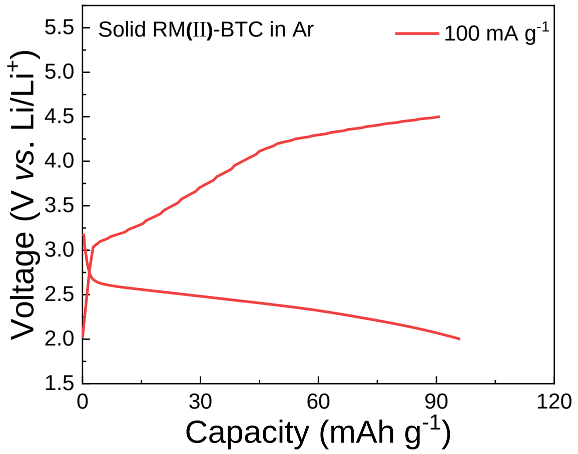

Supplementary Fig. 4. The first Charging/discharging curves of solid RM(II)-BTC cathode in Ar atmosphere at 100 mA g^{-1}.

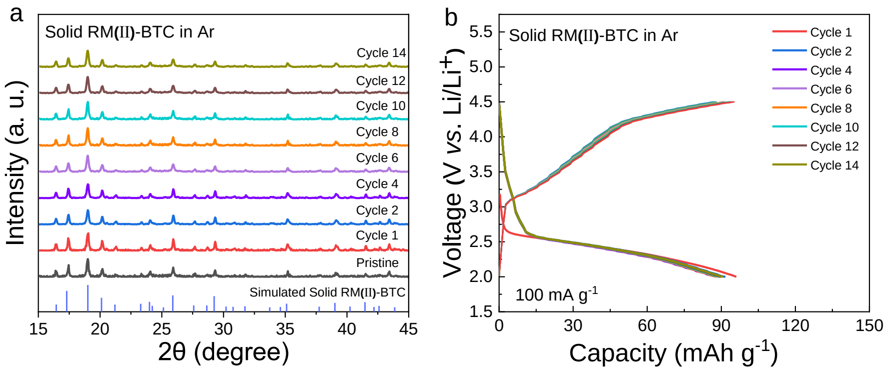

Supplementary Fig. 5. (a) In-situ XRD spectra of solid RM(II)-BTC cathode during charging and discharging in Ar. (b) The charging/discharging curves of solid RM(II)-BTC cathode in Ar at 100 mA g^{-1}.

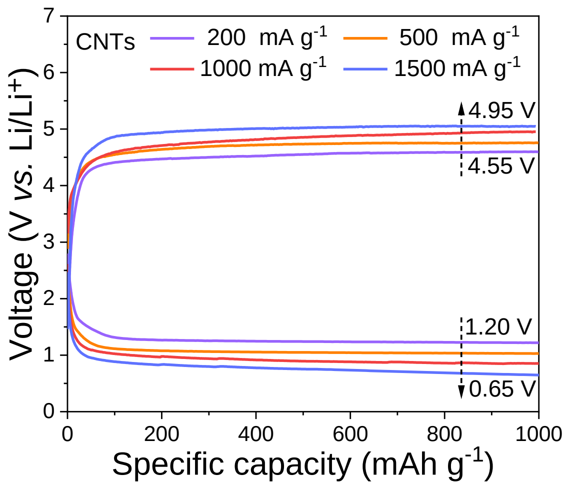

Supplementary Fig. 6. Charging/discharging curves of CNTs cathode with a fixed capacity of 1000 mAh g^{-1} at various current density.

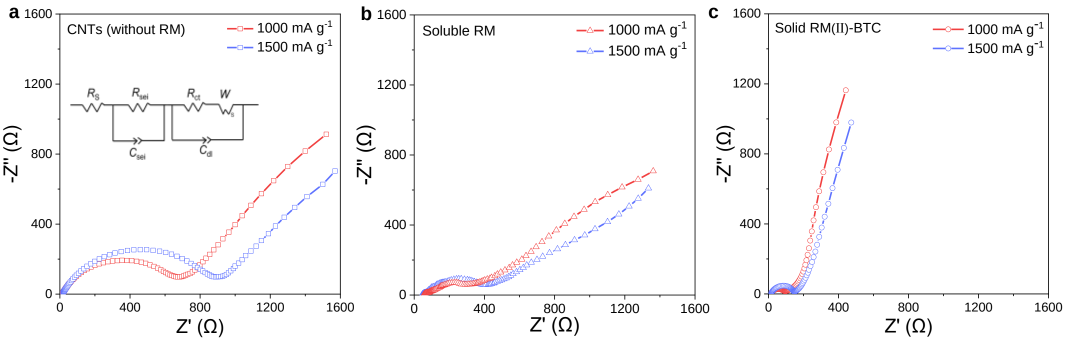

Supplementary Fig. 7. The EIS spectra of the CNTs (without RM), soluble RM and solid RM(II)-BTC at various current density with a fixed capacity (1000 mAh g^{-1}). Insets: an equivalent circuit is used to fit the EIS data. R_s reflects the ohmic resistance that comprises contributions from the electrolyte, electrodes, current leads, and so on. R_{sei} denotes the resistance of solid electrolyte interfacial layers on the air electrode, whereas C_{sei} represents its capacitance. Similarly, R_{ct} and C_{dl} are attributable to charge-transfer resistance and double-layer capacitance, respectively. W represents the Warburg impedance, that arises from a diffusion process.

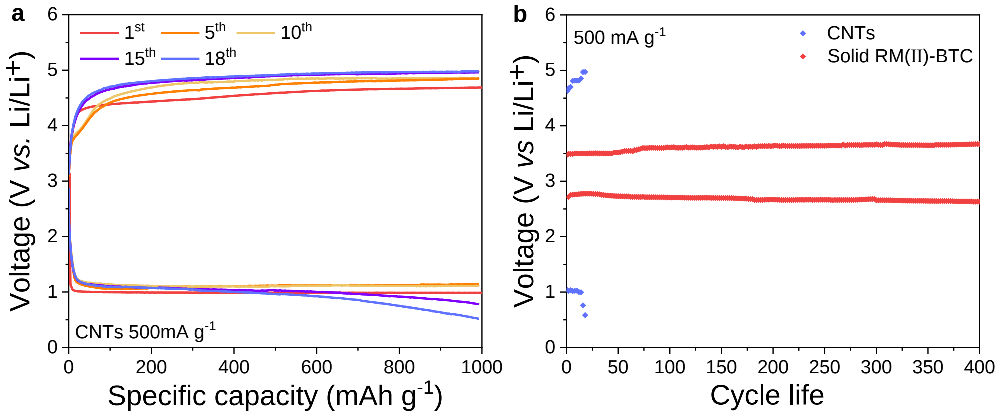

Supplementary Fig. 8. (a) Cycling behavior of CNTs cathode for the selected cycles under CO_2 at a current of 500 mA g^{-1} and curtailing capacity of 1000 mAh g^{-1}. (b) Cycling stability of CNTs and solid RM(II)-BTC cathode at a large current density of 500 mA g^{-1} with fixed capacity of 1000 mAh g^{-1}.

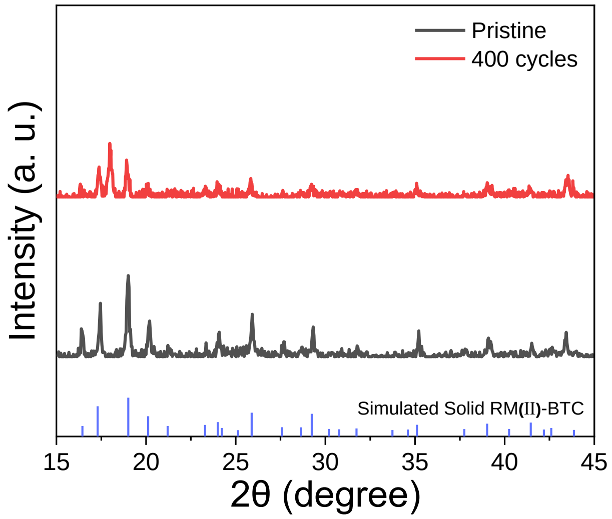

Supplementary Fig. 9. XRD results of solid RM(II)-BTC cathode at pristine and 400 cycles.

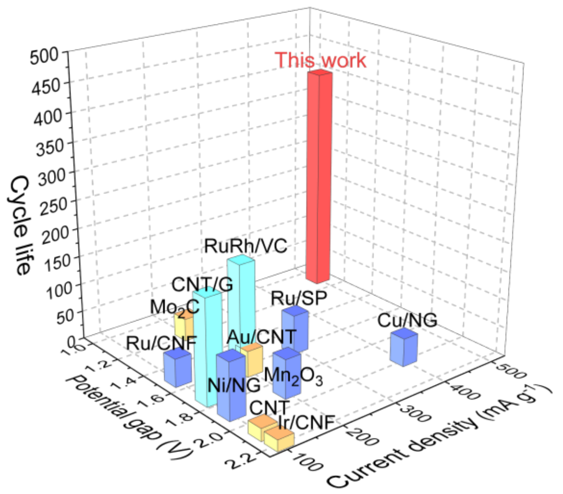

Supplementary Fig. 10. The comparison of different cathode materials for Li-CO_2 batteries in the aspects of current density, potential gap, and cycle number with a fixed specific capacity (1000 mAh g^{-1}).

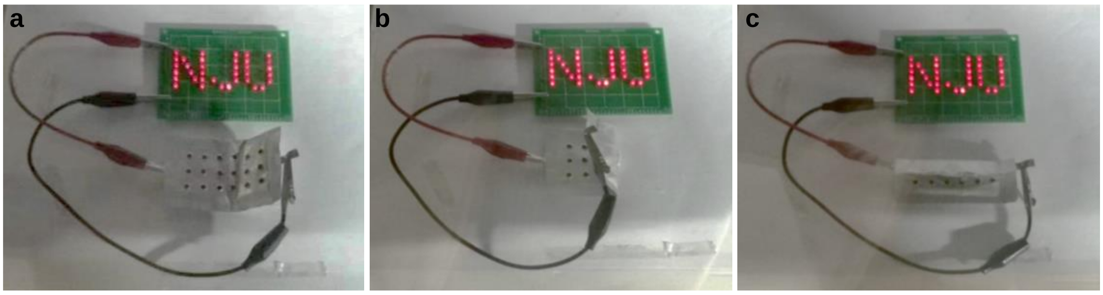

Supplementary Fig. 11. The fabricated pouch battery of solid RM(II)-BTC cathode provides power output for red LED lights even at varied bending angles ranging from 60°(a) to 90° (b) and 180° (c).

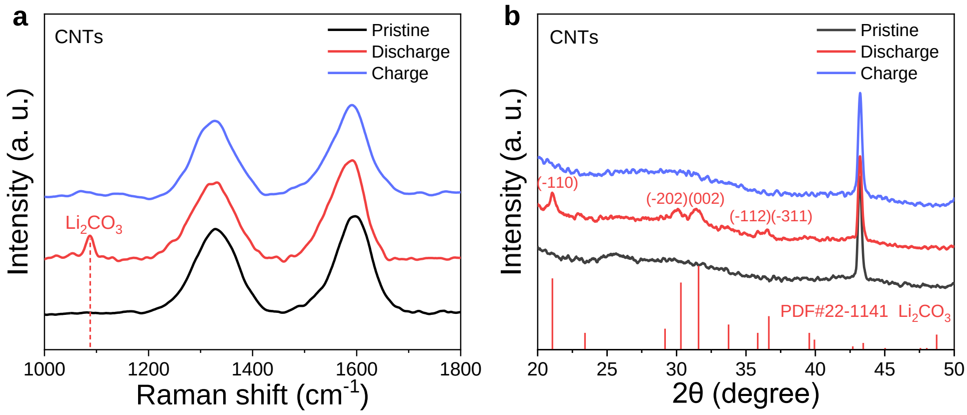

Supplementary Fig. 12. Raman spectrums (a) and XRD patterns (b) of Li-CO_2 battery using the CNTs cathode at different stages (pristine, discharge, recharge).

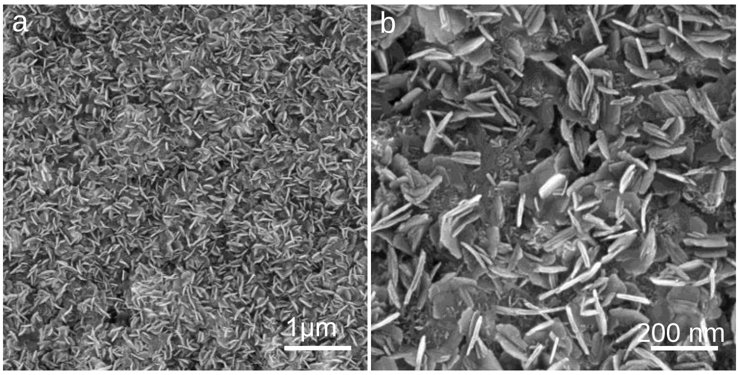

Supplementary Fig. 13. SEM images of CNTs cathode at discharge stage. (a) scale bar = 1μm, (b) scale bar = 200 nm.

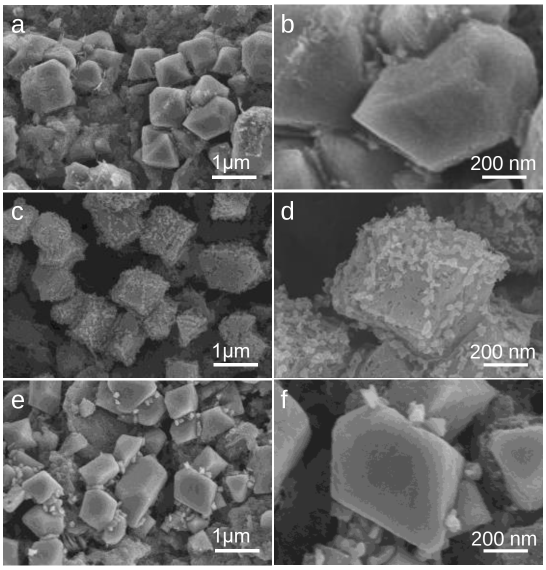

Supplementary Fig. 14. SEM images of solid RM(II)-BTC cathode at different stages (a, b) pristine, (c, d) discharge, (e, f) recharge. (a, c, e) scale bar = 1 μm, (b, d, f) scale bar = 200 nm.

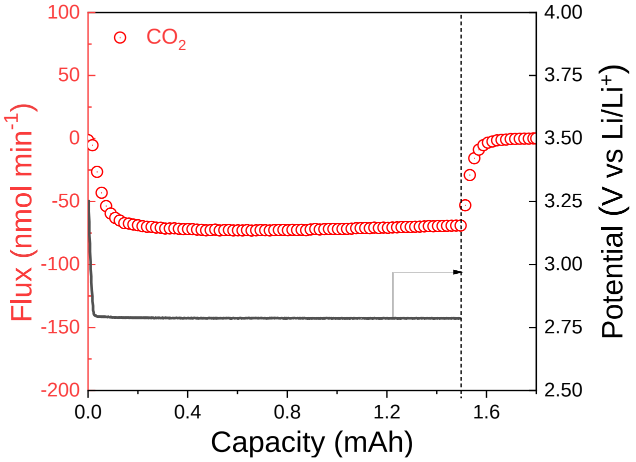

Supplementary Fig. 15. DEMS test during discharging of Li-CO_2 battery using solid RM(II)-BTC cathode.

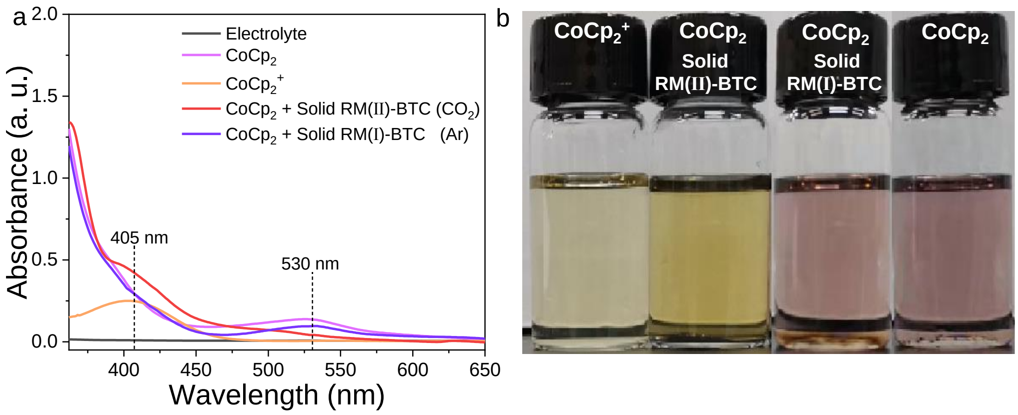

Supplementary Fig. 16. UV-visible spectra (a) and photograph (b) showing the changes upon addition of the reduction product of solid RM(II)-BTC cathode in CO_2 (Cu(II)) or Ar (Cu(I)) to the solution containing $CoCp_2$.

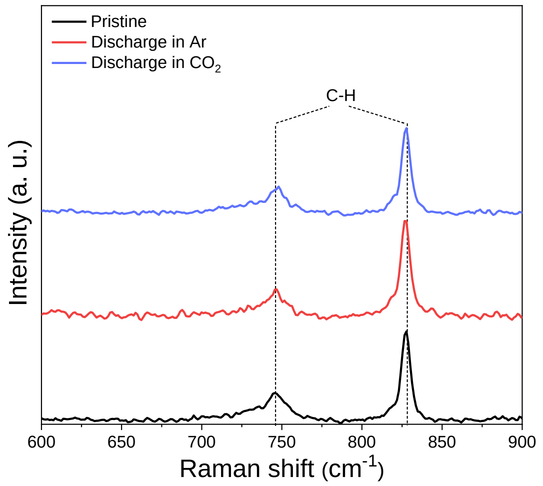

Supplementary Fig. 17. Raman spectra of solid RM(II)-BTC cathode in the Li-CO_2 battery at different stages.

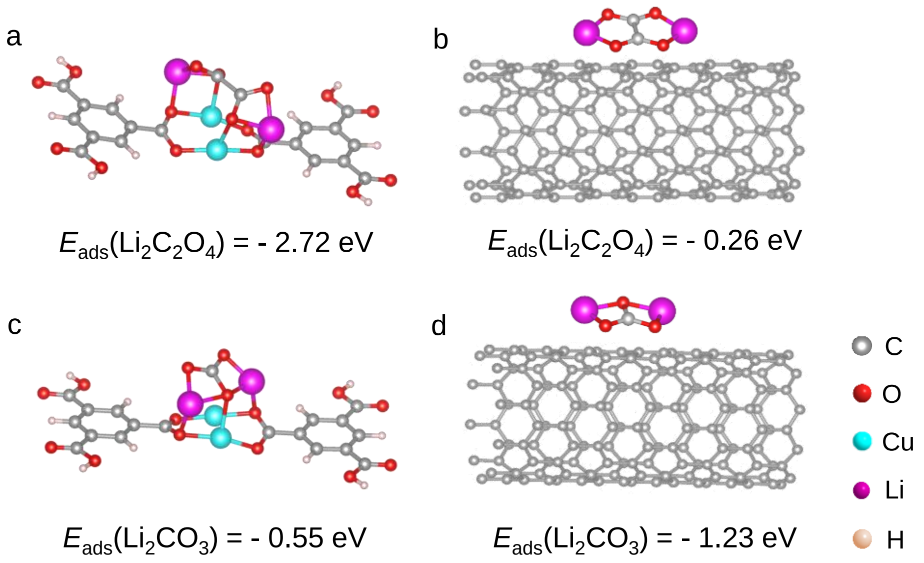

Supplementary Fig. 18. First-principles calculations on basis of the density functional theory (DFT). $Li_2C_2O_4$ adsorbed on (a) solid RM(II)-BTC and (b) CNTs surface. Li_2CO_3 adsorbed on (c) solid RM(II)-BTC and (d) CNTs surface. The grey, red, cyan, purple and light orange balls represent C, O, Cu, Li and H, respectively.

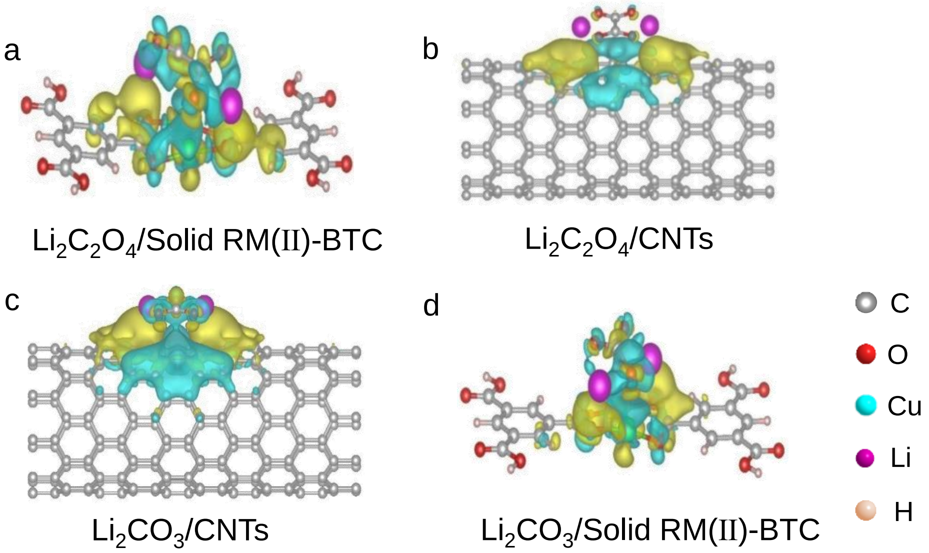

Supplementary Fig. 19. Charge density difference of $Li_2C_2O_4$ adsorbed on solid RM(II)-BTC (a) surface and CNTs (b) surface. Charge density difference of Li_2CO_3 adsorbed on CNTs (c) surface and solid RM(II)-BTC (d) surface. The grey, red, cyan, purple and light orange balls represent C, O, Cu, Li and H, respectively.

Supplementary Table 1 Circuit values with different catalysts at a fixed capacity (1000 mAh g^{-1}).

Catalysts	Current	Mass loading	R_t (Ω)	R_s (Ω)	R_{int} (Ω)	C_{int} (μF)	C_{dl}(μF)	Error (%)
CNTs	1000 mA g^{-1}	0.3±0.05 mg	670.5	15.6	22.6	0.34	18.2	4.76
	1500 mA g^{-1}	0.3±0.05 mg	965.2	17.2	23.7	0.36	19.3	4.68
Soluble RM	1000mA g^{-1}	0.3±0.05 mg	260.6	48.5	21.3	0.29	14.4	4.53
	1500mA g^{-1}	0.3±0.05 mg	420.7	50.2	22.4	0.31	15.1	4.69
RM(II)-BTC	1000mA g^{-1}	0.3±0.05 mg	110.4	14.8	21.1	0.27	7.3	4.43
	1500mA g^{-1}	0.3±0.05 mg	140.8	15.6	22.5	0.29	7.9	4.64

Supplementary Table 2 Summary of recent research progress in Li-CO_2 batteries with carbon-based catalysts.

Ref	Cathode	current density/ cut-off capacity	Mass loading	Discharge/charge voltage	Cycling life
[1]	UiO-66	50 mA g^{-1} 1000 mAh g^{-1}	0.3–0.5 mg	2.75 V/4.50 V	20 cycles
[2]	UiO-66-NH_2	100 mA g^{-1} 1000 mAh g^{-1}	/	2.50V/4.60V	22 cycles
[3]	ZIF-8	1000 mA g^{-1} 1000 mAh g^{-1}	0.3 mg	2.40 V/4.50 V	50 cycles
[4]	ZIF-67	1000 mA g^{-1} 1000 mAh g^{-1}	0.1 mg	2.00 V/4.50 V	140 cycles
[5]	Fe-MIL-NH_2	100 mA g^{-1} 1000 mAh g^{-1}	0.35 mg	2.50 V/4.25 V	185 cycles
[6]	IR-MOF-3	200 mA g^{-1} 500 mAh g^{-1}	0.2 mg	2.25V/4.50 V	538 cycles
[7]	CPO-27-Zn	250 mA g^{-1} 1000 mAh g^{-1}	0.051 mg	2.50 V/4.30 V	120 cycles

Supplementary Table 3 Summary of recent research progress in Li-CO_2 batteries with noble metal catalysts.

Ref	Cathode	current density/ cut-off capacity	Mass loading	Discharge/charge voltage	Cycling life
[8]	Ru@Super P	300 mA g^{-1} 1000 mAh g^{-1}	0.2–0.3 mg	2.50 V/4.15V	70 cycles
[9]	Ir/CNFs	100 mA g^{-1} 1000 mAh g^{-1}	0.2 mg	2.40V/4.60V	20 cycles
[10]	Ru/CNF	100 mA g^{-1} 1000 mAh g^{-1}	0.08–0.12 mg	2.70 V/4.25 V	50 cycles
[11]	CNT@RuO_2	50 mA g^{-1} 500 mAh g^{-1}	0.3–0.5 mg	2.50 V/4.25 V	55 cycles
[12]	RuRh NS /VC72	200 mA g^{-1} 1000 mAh g^{-1}	0.2–0.4 mg	2.30 V/3.90 V	190 cycles
[13]	Ru/CNT	100 mA g^{-1} 500 mAh g^{-1}	0.2 mg	2.50V/4.10 V	100 cycles
[14]	IrO_2-N/CNT	100 mA g^{-1} 400 mAh g^{-1}	0.2–0.3 mg	2.60 V/3.95 V	316 cycles
[15]	Au/CNTs	200 mA g^{-1} 1000 mAh g^{-1}	0.5 mg	2.35 V/4.50 V	46 cycles

Supplementary Table 4 Summary of recent research progress in Li-CO_2 batteries with transition metal compound catalysts.

Ref	Cathode	current density/<br>cut-off capacity	Mass loading	Discharge/charge voltage	Cycling life
[16]	Ni-NG	100 mA g^{-1}<br>1000 mAh g^{-1}	0.3–0.5 mg	2.50 V/4.40 V	101 cycles
[17]	Cu-NG	400 mA g^{-1}<br>1000 mAh g^{-1}	0.3–0.5 mg	2.30 V/4.30 V	50 cycles
[18]	adjacent Co/GO	50 mA g^{-1}<br>1000 mAh g^{-1}	0.9-1.1 mg	2.35 V/4.35 V	100 cycles
[19]	Fe-ISA/N, S-HG	1000 mA g^{-1}<br>1000 mAh g^{-1}	0.2 mg	2.50 V/4.40 V	100 cycles
[20]	NiO-CNTs	50 mA g^{-1}<br>500 mAh g^{-1}	/	2.40 V/4.50 V	25 cycles
[21]	Mn_2O_3-Mn_3O_4	200 mA g^{-1}<br>1000 mAh g^{-1}	0.2 mg	2.60 V/4.50	69 cycles
[22]	$ZnCo_2O_4$@CNT	100 mA g^{-1}<br>500 mAh g^{-1}	0.5 mg	2.50 V/4.25 V	130 cycles
[23]	VN-NW GDC	100 mA g^{-1}<br>400 mAh g^{-1}	0.3 mg	2.80 V/3.70 V	100 cycles
[24]	Mo_2C/CNT	200 mA g^{-1}<br>1000 mAh g^{-1}	3 mg	2.50 V/3.70 V	40 cycles
[25]	MoS_2/CNT	100 mA g^{-1}<br>400 mAh g^{-1}	/	2.15 V/4.25 V	142 cycles

Supplementary methods

Charge-to-mass calculation of differential electrochemical mass spectrometry. In-situ DEMS was conducted to verify the speculation by measuring transferred electrons and generated CO_2 during the charge process. The practical value of electrons (n_e) based on the charge capacity (Q_{the}) can be calculated as follows:

Q_{the} = It = 0.15 mA×10 h = 1.5 mAh = 1.5×10^{-3}A×3600s = 5.4 C

$$n_e = \frac{Q_{the}}{Q_e \times N_A} = \frac{5.4\ \mathrm{C}}{1.602\times10^{-19}\mathrm{C}\times6.02\times10^{23}\ \mathrm{mol}^{-1}} = 55.99\ \mu\mathrm{mol}$$

$$\frac{\mathrm{Z}}{\mathrm{m}} = \frac{n_e}{n_{CO2}} = \frac{55.99\ \mu\mathrm{mol}}{53.32\ \ \mu\mathrm{mol}} = 1.05$$

Based on the CO_2 evolution quantity (n_{CO2}, 53.32 μmol), the practical charge-to-mass ratio is 1.05 close to 1e^-/CO_2, confirming the CO_2 evolution based on equation $Li_2C_2O_4 \rightarrow 2Li^+ + 2e^- + 2CO_2$.

References

1. Zhang, Z. et al. The first introduction of graphene to rechargeable Li-CO_2 batteries. *Angew. Chem. Int. Ed.* **54**, 6550-6553 (2015).
2. Zhang, X. et al. Rechargeable Li-CO_2 batteries with carbon nanotubes as air cathodes. *Chem. Commun.* **51**, 14636-14639 (2015).
3. Qie, L. et al. Highly rechargeable lithium-CO_2 batteries with a boron- and nitrogen-codoped holey-graphene cathode. *Angew. Chem. Int. Ed.* **56**, 6970-6974 (2017).
4. Jin, Y. et al. High-performance Li-CO_2 batteries based on metal-free carbon quantum dot/holey graphene composite catalysts. *Adv. Funct. Mater.* **28**, 1804630 (2018).
5. Xiao, Y. et al. High-performance Li-CO_2 batteries from free-standing, binder-free, bifunctional three-dimensional carbon catalysts. *ACS Energy Lett.* **5**, 916-921 (2020).
6. Song, L. et al. An ultra-long life, high-performance, flexible Li-CO_2 battery based on multifunctional carbon electrocatalysts. *Nano Energy* **71**, 104595, (2020).
7. Li, X. et al. Vertically aligned N-doped carbon nanotubes arrays as efficient binder-free catalysts for flexible Li-CO_2 batteries. *Energy Storage Mater.* **35**, 148-156 (2021).
8. Yang, S. X. et al. A reversible lithium-CO_2 battery with Ru nanoparticles as a cathode catalyst. *Energy Environ. Sci.* **10**, 972-978 (2017).
9. Wang, C. et al. Fabricating Ir/C nanofiber networks as free-standing air cathodes for rechargeable Li-CO_2 batteries. *Small* **14**, 1800641 (2018).
10. Qiao, Y. et al. Transient, in situ synthesis of ultrafine ruthenium nanoparticles for a high-rate Li-CO_2 battery. *Energy Environ. Sci.* **12**, 1100-1107 (2019).
11. Bie, S. et al. Carbon nanotube@RuO_2 as a high performance catalyst for Li-CO_2 batteries. *ACS Appl. Mater. Interfaces* **11**, 5146-5151 (2019).
12. Xing, Y. et al. Ultrathin RuRh alloy nanosheets enable high-performance lithium-CO_2 battery. *Matter* **2**, 1494-1508 (2020).
13. Thoka, S. et al. Comparative study of Li-CO_2 and Na-CO_2 batteries with Ru@CNT as a cathode catalyst. *ACS Appl. Mater. Interfaces* **13**, 480-490 (2021).
14. Wu, G. et al. Design of ultralong-life Li-CO_2 batteries with IrO_2 nanoparticles highly dispersed on nitrogen-doped carbon nanotubes. *J. Mater. Chem. A* **8**, 3763-3770 (2020).
15. Kong, Y. et al. Nano-sized Au particle-modified carbon nanotubes as an effective and stable cathode for Li-CO_2 batteries. *Eur. J. Inorg.Chem.* **6**, 590-596 (2021).
16. Zhang, Z. et al. Verifying the rechargeability of Li-CO_2 batteries on working cathodes of Ni nanoparticles highly dispersed on N-doped graphene. *Adv. Sci.* **5**, 1700567 (2018).
17. Zhang, Z. et al. Identification of cathode stability in Li-CO_2 batteries with Cu nanoparticles highly dispersed on N-doped graphene. *J. Mater. Chem. A* **6**, 3218-3223 (2018).
18. Zhang, B. W. et al. Targeted synergy between adjacent Co atoms on graphene oxide as an efficient new electrocatalyst for Li-CO_2 batteries. *Adv. Funct. Mater.* **29**, 1904206 (2019).
19. Hu, C. et al. High-performance, long-life, rechargeable Li-CO_2 batteries based on a 3D holey graphene cathode implanted with single iron atoms. *Adv. Mater.* **32**, 1907436 (2020).
20. Zhang, X. et al. High performance Li-CO_2 batteries with NiO-CNT cathodes. *J. Mater. Chem. A* **6**, 2792-2796 (2018).

21. Liu, L. et al. Understanding the dual-phase synergy mechanism in Mn_2O_3-Mn_3O_4 catalyst for efficient Li-CO_2 batteries. *ACS Appl. Mater. Interfaces* **12**, 33846-33854 (2020).
22. Thoka, S. et al. Spinel zinc cobalt oxide ($ZnCo_2O_4$) porous nanorods as a cathode material for highly durable Li-CO_2 batteries. *ACS Appl. Mater. Interfaces* **12**, 17353-17363 (2020).
23. Pipes, R., He, J., Bhargav, A., Manthiram, A. Freestanding vanadium nitride nanowire membrane as an efficient, carbon-free gas diffusion cathode for Li-CO_2 batteries. *Energy Storage Mater.* **31**, 95-104 (2020).
24. Hou, Y. et al. Mo_2C/CNT: An efficient catalyst for rechargeable Li-CO_2 batteries. *Adv. Funct. Mater.* **27**, 1700564 (2017) .
25. Chen, C. J. et al. Catalytically active site identification of molybdenum disulfide as gas cathode in a nonaqueous Li-CO_2 battery. *ACS Appl. Mater. Interfaces* **13**, 6156-6167 (2021).
